# Conditional neuronal deletion of microRNA‐141/200c cluster, but not microRNA‐181a/b‐1 cluster, is protective against experimental stroke in male mice

**DOI:** 10.14814/phy2.70505

**Published:** 2025-08-14

**Authors:** Lijun Xu, Brian Griffiths, Xiaoyun Sun, Majesty Greer, Heather Chang, Elijah McMillin, Elizabeth Manis, Isabella Russo, Claire Ai Jue Dean, Creed M. Stary

**Affiliations:** ^1^ Department of Anesthesiology, Perioperative and Pain Medicine Stanford University, School of Medicine Palo Alto California USA

**Keywords:** cell type, CKIIα, MCAO, miR, miRNA

## Abstract

MicroRNAs (miRs) regulate the translation of target genes often in a cell‐type specific manner. We previously demonstrated that downregulation of either miR‐181a or miR‐200c with intracranial injection of an inhibitor is protective against experimental stroke in mice. Here, we generated genetic lines of inducible Ca^2+^‐calmodulin kinase IIα (CKIIα) neuronal miR‐181a/b‐1 and miR‐141/200c cluster deletion to investigate whether the protective effect of their inhibition could be neuron‐specific. Jackson Lab strains Mirc14tm1.1Czc/J and Mirc13tm1Mtm/Mmjax were each crossed with the tamoxifen‐inducible Cre‐recombinase strain B6;129S6‐Tg, CKIIα‐cre/ERT2. Adult double transgenic male mice were randomized and treated with 3 mg tamoxifen or vehicle via oral gavage for 7 days prior to 1 h middle cerebral artery occlusion (MCAO) or sham surgery. Mice were assessed for gross motor function at 24 h and then sacrificed for quantification of infarct volume. Separate animals were assessed for cell‐type specific brain expression of miR‐181a and miR‐200c via combined fluorescent immunohistochemistry and in situ hybridization. Brains from tamoxifen treated mice exhibited selective miR deletion in CKIIα neurons. Infarct volumes were significantly lower, and neurological scores significantly improved in CKIIα/miR‐200c mice pretreated with tamoxifen versus vehicle alone. In contrast, no difference was observed in infarct volume or neurological score in CKIIα/miR‐181a mice pretreated with tamoxifen versus vehicle.

## 1 INTRODUCTION

Stroke remains a leading source of mortality and disability worldwide. Despite promising preclinical trials of gene therapies, pharmaceutical therapy remains limited to early reperfusion with thrombolytics. MicroRNAs (miRs) are noncoding RNAs that regulate the translation of target genes, with a growing body of evidence supporting a role for miRs as a novel stroke therapy. Endogenous expression of miRs can be manipulated through exogenous application of miR mimics and inhibitors, and the translational success of miR‐based therapeutics in preclinical models has led to the successful development of pharmaceutical therapies for hepatitis C (Janssen et al., 2013) and liver cancer (Bader, 2012). Cerebral ischemia has been reported to alter the expression of more than 20% of brain miRNAs (Bhalala et al., 2013; Saito & Saito, 2012), suggesting a central role for miRs as potential targets to mitigate the evolution of injury after stroke. We have previously demonstrated that downregulation of either miR‐181a (Ouyang et al., 2012) or miR‐200c (Stary et al., 2015) in the brain using intracranial injection of miR inhibitors was protective against experimental stroke in mice. We further demonstrated that intravenous injection of miR‐181a (Xu et al., 2015) and miR‐200c inhibitors (Griffiths et al., 2022) was also effective in providing protection against cerebral ischemia. However, systemic application of miR‐based therapies raises concerns for off‐target effects, and indeed our prior work (Leng et al., 2023) and others (Liu et al., 2021; Rogg et al., 2018) have demonstrated that miR biology can have both cell‐type and tissue‐specific effects. Therefore, investigations are needed to determine the cell‐type specific roles of miRs in injury progression to advance novel targeted miR‐based therapies for stroke.

Calcium/calmodulin‐dependent protein kinase II (CKII) is found abundantly throughout the CNS (Miller et al., 2017), where it serves to regulate calcium signaling via phosphorylation of various critical proteins, including neuronal membrane receptors and intracellular transcription factors. Prior work (Miller et al., 2017; Ochiishi et al., 1994) confirmed that the CKIIα isoform is localized only in neuronal elements and is abundantly expressed in the forebrain within the distribution of the middle cerebral artery, the most frequently occluded cerebral vessel in clinical stroke. To build on our prior work and establish whether miR‐181a or miR‐200c play a cell‐specific role in stroke injury, we generated genetic cre/lox mouse lines of tamoxifen‐inducible miR‐181a/b‐1 and miR‐141/200c cluster deletion under the control of the CKIIα promoter. After CKIIα–dependent miR deletion with tamoxifen treatment, we then subjected these lines to experimental transient focal cerebral ischemia to investigate whether the protective effect of miR inhibition was CKIIα neuron‐dependent.

## 2 METHODS

### 2.1 Generation of inducible neuron‐specific miR knockdown mice

The present study was conducted in accordance with National Institutes of Health guidelines for the use of experimental animals. All protocols were approved by the Stanford Animal Care and Use Committee (Assurance Number A3213‐01). Animals were kept in air‐conditioned rooms in a controlled environment at 21 ± 2°C with a 12 h/12 h light and darkness cycle and fed standard diets (#5001, LabDiet). Jackson Lab strains *Mirc14* ^*tm1.1Czc*^/J, miR‐181a/b‐1 cluster (Fragoso et al., 2012), and *Mirc13* ^*tm1Mtm*^/Mmjax, miR‐141/200c cluster (Park et al., 2012) were purchased and crossed with the tamoxifen‐inducible Cre‐recombinase strain B6;129S6‐Tg, CKIIα‐cre/ERT2 (Madisen et al., 2010), under the control of the neuronal CKIIα promoter. Adult male (8–10 weeks old) double transgenic mice and wildtype littermates were identified by tail genotyping (TransnetYX Inc.). To induce miR deletion, animals were randomized to treatment with 3 mg tamoxifen (#10540‐29‐1, ThermoFisher Scientific) or pharmaceutical grade sesame seed oil (“vehicle,” #8008‐74‐0, ThermoFisher Scientific) alone via oral gavage for 7 days (Whitfield et al., 2015).

### 2.2 Fluorescent in situ hybridization and immunohistochemistry

To determine qualitative cell‐type specific and regional expression of miR‐181a and miR‐200c, we complexed fluorescent in situ hybridization (FISH) with fluorescent immunohistochemistry as previously described (Arvola et al., 2019, 2021). After 7 days treatment with tamoxifen or oil, animals were sacrificed, and sections from the hippocampal plane (−1.2 to −2.0 mm from anterior commissure) were isolated for staining. Sections were incubated in primary antibody solutions consisting of rabbit‐NeuN ([1:500] #NBP1‐77686, Novus Biologicals) and chicken‐GFAP ([1:500] #ab4674, Abcam) followed by secondary antibody incubation with donkey anti‐chicken Alexa Fluor 647 ([1:500] #703‐606‐155, Jackson Immunoresearch Laboratories Inc), donkey‐anti‐rabbit Alexa Fluor 594 ([1:500] #A21207, Abcam), and 4′,6‐diamidino‐2‐phenylindole‐hydrochloride (DAPI, [1:1000] #1700624 Life Technologies). Sections were then incubated with FAM‐labeled miR‐181a‐5p (#YD00619309‐BED, Qiagen) or miR‐200c‐5p (#YD00611801‐BED, Qiagen) miRCURY LNA miRNA detection probes as described previously (Arvola et al., 2019, 2021). Small nuclear RNA U6 was used as a positive control, and a scrambled probe with no complementarity to any known miRNAs was used as a negative control as previously described (Arvola et al., 2019, 2021). All sections were imaged using a Zeiss Axio Imager M2 (Carl Zeiss AG) with Apotome 2.0 with Neurolucida (MBF Bioscience).

### 2.3 Transient focal cerebral ischemia

Transient middle cerebral artery occlusion (MCAO) was used to model ischemic stroke as previously described (Xu et al., 2015). Briefly, after 7 days treatment with tamoxifen or oil, mice were anesthetized with 1.5%–2% isoflurane, and a skin incision was made in the neck to expose the internal carotid artery. Cerebral ischemia was induced with a silicone‐coated 6‐monofilament (Doccol Co) introduced in the internal carotid artery and advanced into the middle cerebral artery (MCA). During the surgical procedure, rectal temperature was maintained at 37 ± 0.5°C with a homeothermic blanket control unit (Harvard Apparatus) and cerebral blood flow was monitored with a laser Doppler (Moor Instruments). As MCAO primarily affects the motor cortex, gross motor function (neurological deficit score) was assessed after 24 h of reperfusion as previously described (Yang et al., 1994) at 24 h prior to sacrifice by isoflurane overdose. Neurological deficit score was graded as follows: (0) no deficit, (1) forelimb weakness, failure to extend forepaw; (2) torso turning to the ipsilateral side when held by tail, circling to the affected side; (3) inability to bear weight on the affected side, falling; (4) no spontaneous locomotor activity.

### 2.4 Calculation of infarct volume

Mice were sacrificed 24 h after MCAO with isoflurane overdose, a time‐point that coincides with the peak of stroke injury (Liu et al., 2009). After sacrifice, mice were cardiac perfused with ice‐cold saline, and brains were removed and sliced into four sections/brain using a steel coronal matrix (Roboz Surgical Instrument Co.). Slices were then incubated with 2,3,5 triphenyltetrazolium chloride (TTC, Sigma, #T8877) at 37°C and analyzed by using ImageJ software (National Institutes of Health, Bethesda). The analysis process was conducted by an observer blinded to the treatment group. Infarct volume was corrected for edema using the method by Swanson et al. (1990) by assessing the difference in volume between the contralateral hemisphere and the non‐infarcted region of the ipsilateral hemisphere to arrive at an absolute volume of the infarct tissue.

### 2.5 Statistical analysis

IBM SPSS Statistics 24 (version 24 64‐bit edition, IBM) was used for statistical analyses. All data reported are mean ± standard error of the mean. Statistical analysis was performed using independent samples *t*‐tests. A *p* value of <0.05 was considered significant.

## 3 RESULTS

### 3.1 Expression of CKIIα, miR‐181a, and miR‐200c in transgenic mice

**FIGURE 1:**
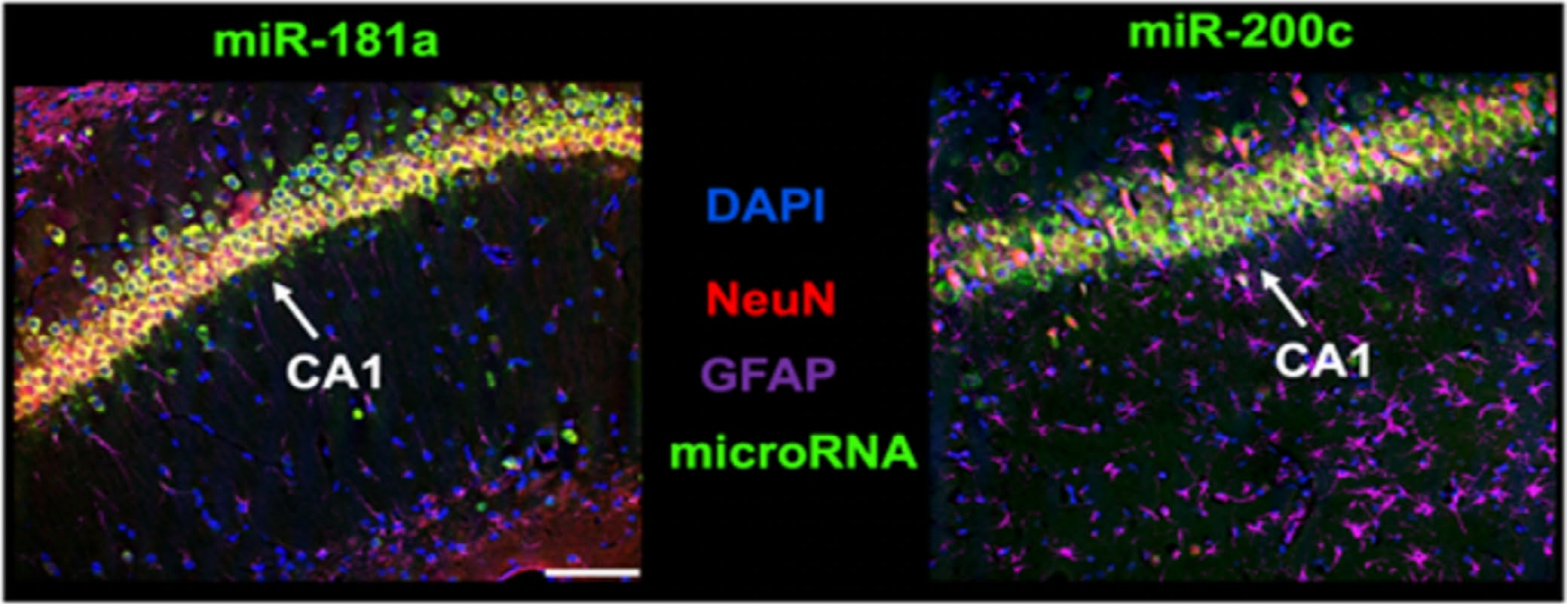
Representative images of fluorescent in situ hybridization (FISH) of miR‐181a (green, left panel) and miR‐200c (green, right panel), complexed with fluorescent immunohistochemistry indicating neurons (NeuN, red), astrocytes (glial fibrillary acidic protein, GFAP, violet), and all cell nuclei (DAPI, blue). Hippocampal cornu ammonis 1 (CA1) is identified as a representative region with high neuronal miR‐181a and miR‐200c expression. Scale bar represents 50 μm.

**FIGURE 2:**
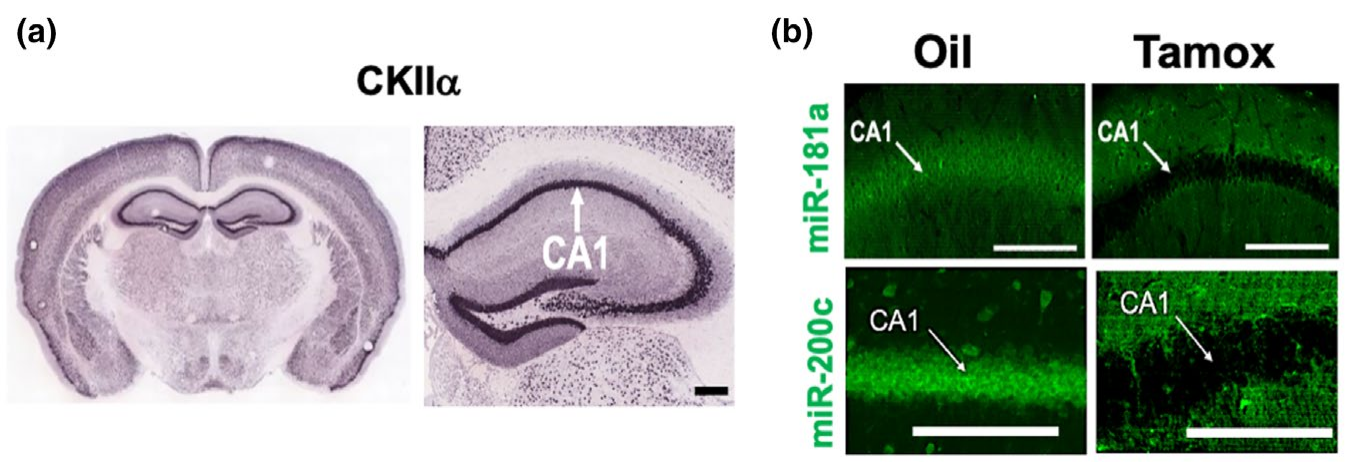
(a) Representative images of in situ hybridization for CKIIα mRNA expression in whole brain (left) and in hippocampus (right) from the Allen Brain Atlas project (Miller et al., 2017). Hippocampal CA1 is identified as a region with high CKIIα mRNA expression. (b) Representative FISH images of hippocampal CA1 in double transgenic CKIIα‐miR‐141/200c and CKIIα‐miR‐181a/b‐1 mice treated with either oil (left) or tamoxifen (right) for 7 days.

Figure 1 demonstrates representative images of the hippocampus in IHC/FISH complexed brains from oil‐treated animals for both miR‐181a and miR‐200c. High levels of expression of miR‐181a and miR‐200c were evident in the neuronal cell layer of cornu ammonis 1 (CA1). In parallel, the hippocampal CA1 subregion has high co‐expression of CKIIα, as demonstrated in a representative image (Figure 2a) from the Allen Brain Atlas (Miller et al., 2017). Figure 2b demonstrates miR‐181a and miR‐200c expression in double transgenic CKIIα‐miR‐181a/b‐1 and CKIIα‐miR‐141/200c mice treated with either tamoxifen or oil for 7 days. Mice treated with tamoxifen exhibited clear evidence of miR‐181a and miR‐200c deletion within the CKIIα‐dense CA1 neuronal layer in CKIIα‐miR‐181a/b‐1 and CKIIα‐miR‐141/200c mice, respectively (Figure 2b).

### 3.2 Infarct volume and neurological deficit in transgenic mice after experimental stroke

**FIGURE 3:**
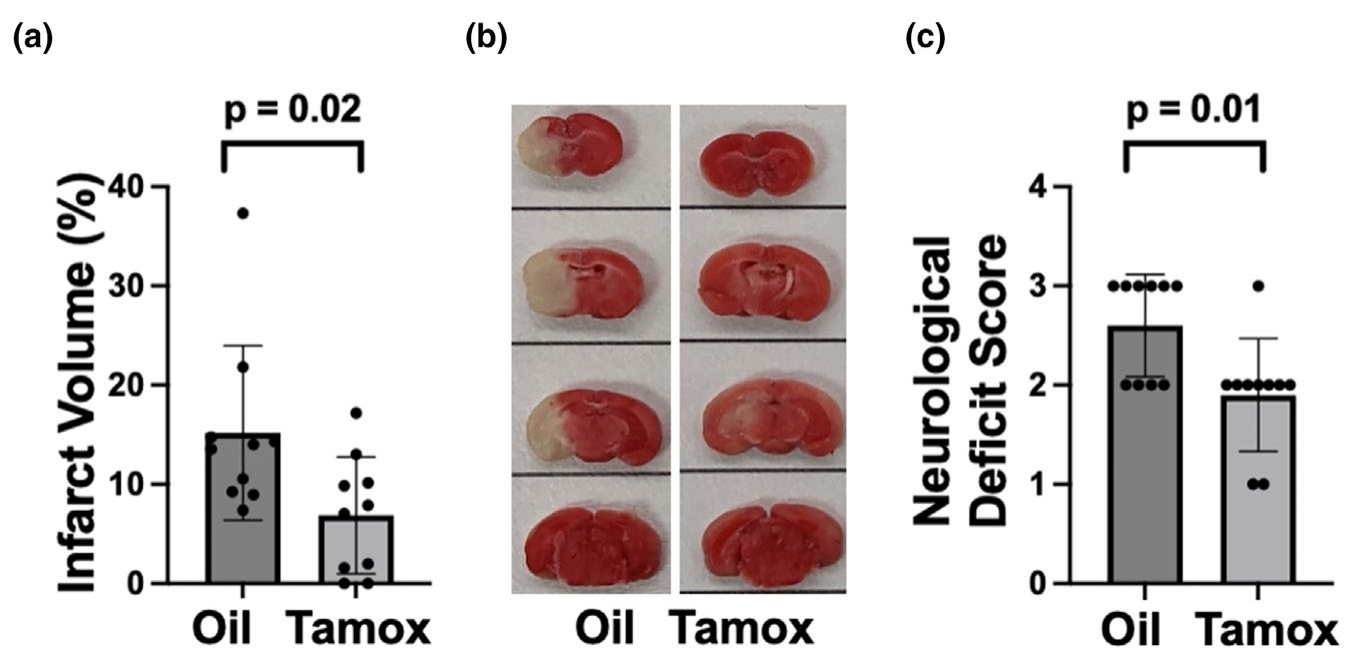
Quantification of infarct volume (a) and a representative TTC‐stained brain (b, white areas represent infarct) 24 h after MCAO in CKIIα‐miR‐141/200c mice pretreated with either oil or tamoxifen for 7 days. (c) Neurological deficit scores 24 h after MCAO. *N* = 10, mean ± SD.

**FIGURE 4:**
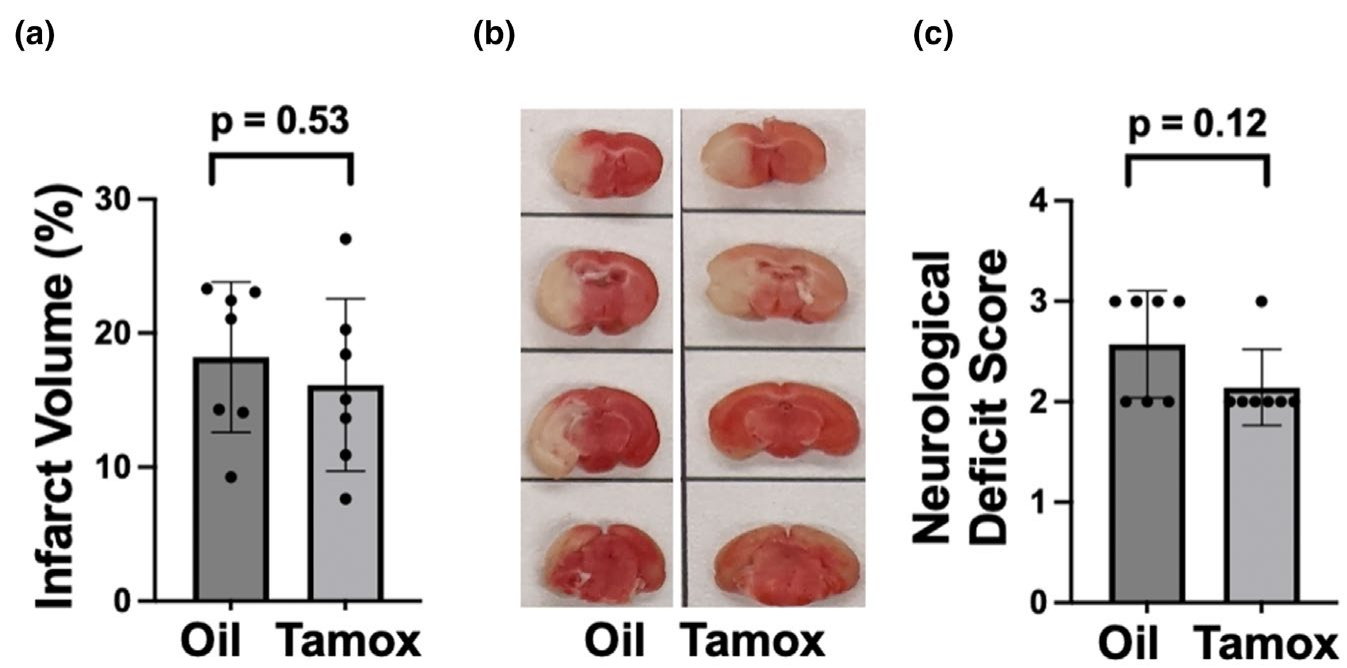
Quantification of infarct volume (a) and a representative TTC‐stained brain (b) 24 h after MCAO in CKIIα‐miR‐181a/b‐1 mice treated with either oil or tamoxifen for 7 days. (c) Neurological deficit scores 24 h after MCAO. *N* = 7, mean ± SD.

To determine whether conditional CKIIα‐dependent deletion of either the miR‐181a/b‐1 cluster or the miR‐141/200c cluster modulates stroke injury, we assessed infarct volume and gross motor function 24 h after 1 h of MCAO in CKIIα‐miR‐181a/b‐1 and CKIIα‐miR‐141/200c mice pretreated with either tamoxifen or oil vehicle. Ipsilateral cerebral blood flow was significantly reduced during MCAO in all treatment groups, with observed between treatments. Infarct volumes were significantly lower and neurological scores significantly improved in CKIIα‐miR‐141/200c mice pretreated with tamoxifen versus treatment with the vehicle alone (Figure 3). In contrast, no difference was observed in infarct volume or in neurological score in CKIIα/miR‐181a/b‐1 mice pretreated with tamoxifen versus the vehicle (Figure 4).

## 4 DISCUSSION

Determining the cell‐type specific effects of miR targeting allows identification of cell‐type specific gene targets for therapy to adjust for off‐target competitive effects, a principal goal of personalized medicine. The results of the present study are the first to definitively demonstrate that cell phenotype can play a dependent role in the biological effect of known miRs relevant to stroke injury whereby conditional (adult) knock out of the neuronal miR‐141/200c cluster was protective against experimental transient focal cerebral ischemia. This result demonstrates that neuronal‐specific miR‐141/200c knockdown is sufficient for protection and not dependent on knockdown in other cell types. Our recent work (Arvola et al., 2021) demonstrated robust expression of miR‐200c after experimental stroke in both astrocytes and neurons. The results from this study suggest that elevations in miR‐200c contribute to stroke injury only when expressed in neurons. This finding is significant in that miR‐200c and downstream mitochondrial targets including sirtuin1, reelin, and Bcl2 have been implicated in both stroke‐related injury and in neuronal‐dependent post‐stroke dementia (vascular dementia or vascular cognitive impairment). Vascular dementia (VaD) accounts for the second most common type of dementia, accounting for approximately 17%–30% of all cases. This result holds substantial relevance toward targeted therapies for this specific post‐stroke manifestation.

Conversely, conditional knockdown of the neuronal miR‐181 cluster did not result in appreciable protection, suggesting that levels of miR‐181a expression play a more significant role in other cell types. MiRs are known to regulate gene expression in a regional and cell‐type specific manner (Pomper et al., 2020), as both a consequence of, and contributing to, cellular phenotype. We previously demonstrated that inhibition of miR‐181a selectively promoted hippocampal neurogenesis after ischemia (Griffiths et al., 2019). Neurons are highly dependent upon astrocytes for their energy requirements. Astrocytes serve many housekeeping functions (Verkhratsky & Nedergaard, 2018) and are indispensable for neuronal homeostasis and in maintaining the supply of phosphorylation equivalents to maintain the high energy demand of neurons (Heneka et al., 2010; Pfrieger, 2009). Neurons consume ~80% of total brain energy (Hyder et al., 2013) to support the restoration of membrane potentials, intracellular vesicular synthesis and packaging, axonal transport, and neurotransmitter synthesis and release (Attwell & Laughlin, 2001; Pathak et al., 2015; Rangaraju et al., 2014). Normal brain activity depends on the metabolic plasticity of astrocytes and requires not only glucose in blood but also glycogen stored in astrocytes (Brown & Ransom, 2007). The results of the present study suggest that miR‐181a/b plays a significant role in an alternative non‐neuronal cell type in stroke injury.

Future studies could utilize alternative Cre‐recombinase promoter strains to identify the functional cellular targets for miR‐181a deletion, and in parallel investigate the direct miR‐200c gene targets in CKIIα‐neurons to advance novel drug discovery for clinical stroke. Few studies to date have investigated the intercellular regulation of CA1 neurodegeneration after global cerebral ischemia (Nemirovich‐Danchenko & Khodanovich, 2019). Zhao et al. (2017) demonstrated that astrocytes act to support neuronal function and repair (Clarke & Barres, 2013) by secreting soluble factors that preserve synaptogenesis in CA1 after ischemia. Recent evidence indicates that astrocytes communicate with neurons via secretion of miRs contained within extracellular vesicles (Budnik et al., 2016; Men et al., 2019; Prada et al., 2018). Using RNA sequencing and microarray hybridization, Merienne et al. (2019) observed a competing miR response to neurodegenerative disease between neurons and glia; however, the role of cell‐type specific miR functions in response to select ischemic CA1 neurodegeneration is unknown. Using combined IHC/FISH, we previously observed augmented CA1 miR‐200c to global cerebral ischemia in reactive astrocytes relative to neurons (Arvola et al., 2019).

Our recent study with miR‐200c in select ischemic CA1 neurodegeneration identified a similar post‐injury response, with increased miR‐200c expression localizing to adjacent regions of reactive astrocytes (Griffiths et al., 2022). Oxidative stress induces regulatory changes in pathways involved in metabolism, apoptosis, ion transport, cell motility, and G‐protein signaling (Oberdoerffer et al., 2008). This complex, multicellular miR‐mediated response to injury can be explored in future studies using single‐cell omics strategies, or alternatively with cell‐type specific in vivo miR silencing, or in vitro studies comparing different cell cultures derived from different hippocampal subregions. One paradoxical limitation of the present study is the ubiquitous expression of CKIIα throughout multiple brain regions. Future investigations could alternatively selectively target‐specific neuronal populations via alternative approaches, for example with CRISPR/Cas9. Another limitation is the deletion of whole clusters of related miR targets versus specific miR 3p and 5p species. Currently, only miR‐141/200c cluster knockout strains are available, with concurrent knockdown of miR‐141 with miR‐200c stem loops, which delete both 5p and 3p species. Clustered miRs tend to be evolutionarily conserved (Wang et al., 2016); for example, miR‐141 shares 6/7 nucleotide sequence homology with the miR‐200c seed sequence. Gene proximity and sequence similarity within clusters therefore provide some biological redundancy in miR regulation of downstream targets (Wang et al., 2016). A final limitation in the present study is the use of only young adult male mice, while our own prior research has demonstrated that miR inhibition can be both sex‐ and age‐dependent (Stary et al., 2017; Xu et al., 2023). Future investigations in clinically relevant aged animals of both sexes are therefore also warranted to isolate the most relevant miR‐based mechanisms for cell‐type specific therapies.

## FUNDING INFORMATION

Supported by American Heart Association grant 18POST33990395 to BGG and NIH grant R01NS107445 to CMS.

## CONFLICT OF INTEREST

None.

## ETHICS STATEMENT

This work is original and has not been published elsewhere, nor is it currently under consideration for publication elsewhere. The authors have no conflicts of interest to declare. The authors have complied with all relevant ethical guidelines for the reporting and publication of research involving animals.

## Data Availability

All data will be made available in part or in whole upon request.
